# Impact of Postoperative Norepinephrine Administration on Free Flap Flow

**DOI:** 10.3390/jcm13247816

**Published:** 2024-12-20

**Authors:** Denis Ehrl, Svenja Pistek, Clemens Rieder, Michael Irlbeck, Klaus Hofmann-Kiefer, David Braig, Frederic Klein, Philipp Groene, Riccardo E. Giunta, Nicholas Moellhoff

**Affiliations:** 1 Division of Hand, Plastic and Aesthetic Surgery, University Hospital, Ludwig-Maximilians-Universität München, 81377 Munich, Germany; svenja.pistek@campus.lmu.de (S.P.); riccardo.giunta@med.uni-muenchen.de (R.E.G.); nicholas.moellhoff@med.uni-muenchen.de (N.M.); 2 Department of Plastic, Reconstructive and Hand Surgery, Burn Center for Severe Burn Injuries, Klinikum Nuremberg Hospital, Paracelsus Medical University, 90419 Nuremberg, Germany; 3 Department of Anaesthesiology, University Hospital, LMU Munich, 81377 Munich, Germany; clemens.rieder@med.uni-muenchen.de (C.R.); michael.irlbeck@med.uni-muenchen.de (M.I.); klaus.hofmann-kiefer@med.uni-muenchen.de (K.H.-K.); philipp.groene@med.uni-muenchen.de (P.G.); 4 Department of Plastic and Hand Surgery, Medical Center—University of Freiburg, Faculty of Medicine, 79106 Freiburg im Breisgau, Germany; david.braig@uniklinik-freiburg.de; 5 www.statistische-Beratung.de, 80339 Munich, Germany; dipl.stat.klein@statistische-beratung.de

**Keywords:** microvascular flow, O2C, vasopressor, hypotension, microsurgery

## Abstract

**Background/Objectives:** The perioperative interplay between blood pressure, vasopressors, and macrocirculation is well established. However, in the context of free flap surgery, the potential impact of these factors on microvascular flow remains elusive. The aim was to evaluate the impact of norepinephrine administration on the microcirculation of free flaps. **Methods:** Postoperative systolic blood pressure (sBP), norepinephrine infusion rates (NIRs), and free flap microcirculation were monitored prospectively and analyzed retrospectively in patients receiving free flap surgery who required postoperative intermediate (IMC) or intensive care (ICU). Blood flow, hemoglobin oxygenation (SO_2_), and relative hemoglobin levels (rHbs) were measured over a period of 24 hours post-anastomosis by laser-doppler flowmetry and white light spectroscopy using the “Oxygen to See” device (O2C, LEA Medizintechnik, Gießen, Germany). Multivariate analysis was performed to determine the impact of NIR on microvascular flow, adjusting for several confounding factors. Subgroup analysis was conducted by categorizing into three groups based on patients’ postoperative sBP. **Results:** Flaps were performed in 105 patients with a mean age of 61.46 ± 16.29 years. Postoperatively, an increase in microvascular flow over time was observed across all free flaps, while NIR decreased and sBP maintained stable values. Multivariate analysis revealed that the time post-anastomosis (B = 3.76, *p* < 0.001), SO_2_ (B = 0.55, *p* < 0.001), rHb (B= −0.79, *p* < 0.001), female gender (B = 29.25, *p* = 0.02), and no previous radiation therapy (B = 41.21, *p* = 0.04) had a significant impact on postoperative microvascular flow in free flaps. NIR, sBP, smoking status, old age, and ASA score showed no significant impact on free flap flow. Further, NIR showed no significant impact on microvascular flow in any of the subgroups investigated. **Conclusions:** These findings support the safety of using norepinephrine for maintaining stable blood pressure without compromising microvascular flow, offering valuable guidance for postoperative management.

## 1. Introduction

Perfusion of organs and tissue is driven by macrocirculation, which distributes oxygenated blood throughout the body. Several physiologic regulatory mechanisms exist to respond to changes in cardiac output or hypovolemia by altering blood pressure and intravasal volume [1]. For example, circulation is controlled by the sympathetic nervous system [2]. In the context of surgery, sympathomimetic drugs (vasopressors) are administered during general anesthesia to counteract hypotension. However, an ongoing discussion surrounds the perioperative use of vasopressors during free flap reconstructions.

Vasopressors enhance cardiac output and vascular resistance and increase contractility and heart rate [3]. While vasopressors elevate systemic blood pressure, which drives macrocirculation and flap perfusion, they can also diminish microcirculation through vasoconstriction, potentially leading to flap ischemia or contributing to thrombosis and, subsequently, flap failure [4,5,6]. Thus, there remains caution in the use of these substances for the management of patients’ hypotension in this specific patient population.

According to a recent literature review on vasopressor administration in patients receiving head and neck reconstructions, no link was found between vasopressor use and flap failure or postoperative complications [7]. Instead, vasopressors are reported to enhance hemodynamic stability, reducing the need for excessive fluid administration and lowering the associated postoperative risks related to postoperative edema [7,8]. However, the authors also conclude that additional studies are required to provide evidence-based recommendations regarding the precise choice, timing, and dosage of vasopressors [7].

One of the main difficulties in establishing a study protocol to investigate the exact impact of vasopressor use and subsequent changes in blood pressure on free flap perfusion is objective and continuous monitoring of microcirculation in free flaps. Our study group has previously established a protocol to continuously monitor free flap microcirculation in the postoperative period utilizing laser-doppler flowmetry and tissue-spectrometry (Oxygen to See (O2C) device, LEA Medizintechnik, Gießen, Germany) [9,10,11], which may be a promising approach to determine the impact of norepinephrine infusion rates (NIR) on free flap microcirculation.

Using the O2C device, the aim of this study was to gain a better understanding of the impact of postoperative NIR and subsequent changes in systolic blood pressure (sBP) on the microvascular flow of free flaps during the early postoperative period.

## 2. Materials and Methods

### 2.1. Study Design

This monocentric study evaluated the impact of postoperative sBP and NIR on microvascular flow in viable free flaps within the first 24 h post-anastomosis. Data were collected prospectively and analyzed retrospectively between 2020 and 2023. Free flaps were performed by a single surgeon (D.E.), to minimize bias from microsurgical technique. Ethical approval was granted by the local Institutional Review Board (IRB protocol number: 20-549, 26 August 2020).

### 2.2. Population

Patients treated in the Division of Hand, Plastic and Aesthetic Surgery of the University Hospital, LMU Munich, were included in this study according to the following criteria: free flap reconstruction with postoperative continuous monitoring of blood pressure (invasive and non-invasive monitoring), norepinephrine infusion rates, and free flap microcirculation in intermediate (IMC) or intensive care units (ICU) at least till the following day post-free flap surgery. Exclusion criteria included incapacity of understanding the aim and scope of this study, minors, and patients who were not monitored in IMC/ICU. As the intention of this study was to evaluate the impact of sBP and norepinephrine rates on microcirculation in viable free flaps, free flaps with major complications (flap loss >10%, total flap loss, urgent operative revision) were also excluded from data analysis. A total of 105 free flaps performed in 105 patients met these criteria and were thus included in this study.

### 2.3. Outcomes and Data Collection

A thorough review of source data, including medical records, surgical reports, premedication records, and physician correspondences, was performed. Demographic information, patient characteristics, perioperative details, and surgical and medical complications were extracted.

### 2.4. Monitoring of Blood Pressure and Norepinephrine Infusion Rates

Patients’ blood pressure (mmHg) was monitored in IMC/ICU non-invasively (cuff pressure) or invasively (arterial line measurements) postoperatively. Data were obtained from patients’ digital medical files and were extracted hourly. Similarly, norepinephrine infusion rates were acquired in mg/h. Postoperative standard of care recommends sBP to remain at approximately 120 mmHg throughout the admission to the IMC/ICU in the early postoperative course. Vasopressors are administered as required to maintain adequate cardiovascular support.

### 2.5. Microcirculation

Microcirculation was evaluated continuously using the O2C device and LFx37 probe (both LEA Medizintechnik, Gießen, Germany). The O2C device employs laser-doppler flowmetry and tissue-spectrometry to measure blood flow (flow), hemoglobin oxygenation (SO_2_), and relative hemoglobin levels (rHb) in the capillary-venous compartment of the vascular tree. Further details were previously described [9,10,11]. The probe was positioned centrally on the distal third of the free flap, specifically distal to the anastomosis, to maximize sensitivity to potential hypoxia. For fixation, double-sided tape was used on fasciocutaneous flaps and skin islands, while the probe was covered with a sterile, vacuum-sealed synthetic cover and directly sutured onto the muscle portion of muscle flaps. Temporary disconnection of the probe was permitted only during patient transportation or wound dressing changes to ensure continuous data monitoring. Mean values of flow, SO_2_, and rHb were extracted hourly using the O2CevaTime software (LEA Medizintechnik, Gießen, Germany).

### 2.6. Statistical Analysis

Statistical analysis was performed by a professional statistician (F.K.). Data are presented as means with standard deviation or as absolute and relative frequencies unless stated otherwise. Normal distribution was assessed using the Shapiro–Wilk test. The Wilcoxon test was used for pairwise comparisons. To determine whether norepinephrine rates had a significant impact on microvascular flow, a multivariate hierarchical mixed effects model was calculated. The following confounding factors were identified as potentially impacting microcirculation and specific blood flow: sBP, smoking status, age, ASA classification, and prior radiation therapy. Further, subgroup analysis was conducted, and patients were categorized into three groups based on their initial sBP values (low sBP: ≤110 mmHg, intermediate sBP: 111–130 mmHg, and 3. high sBP: >130 mmHg) upon presentation to the recovery room (IMC/ICU). All calculations were performed using R Statistical Software (version 4.3.1; R Foundation for Statistical Computing, Vienna, Austria). A *p*-value of 0.05 was set as the level of significance for all statistical tests.

## 3. Results

A total of 105 viable flaps were performed in 105 patients (females: n = 55, 52.4%; males: n = 50, 47.6%) with a mean age of 61.46 ± 16.29 (range: 19–93) and a mean BMI of 25.11 ± 5.76 were included in this study. Patient demographics and operative details are summarized in Table 1 and Table 2. The mean time of surgery was 354.42 ± 110.58 min. Flap ischemia was 48.66 ± 20.39 min on average. Patients were hospitalized for 17.19 ± 7.84 (range: 5–80) days post free-flap surgery, with a mean of 3.97 ± 12.97 (range: 1–45) days on IMC/ICU.

### 3.1. Analysis of Free Flap Microcirculation over Time

Data on microcirculation, blood pressure, and NIR are summarized in Table 3. Microvascular flow showed a significant increase over the 24 h post-anastomosis, with values starting at a mean of 104.27 ± 44.85 AU and reaching 131.63 ± 59.06 AU (*p* < 0.001) 24 h post-anastomosis. Values for SO_2_ and rHb remained constant and showed no significant differences over time. While NIR decreased significantly from a mean of 0.28 ± 0.37 mg/h to 0.06 ± 0.18 mg/h, sBP was stable and showed no significant differences in the overall patient population.

**Figure 1:**
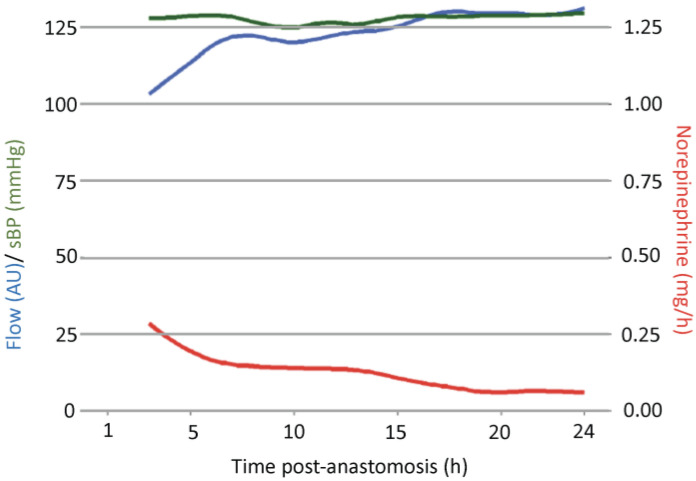
Development of microvascular flow, systolic blood pressure, and norepinephrine infusion rate over 24 h post-anastomosis.

**Figure 2:**
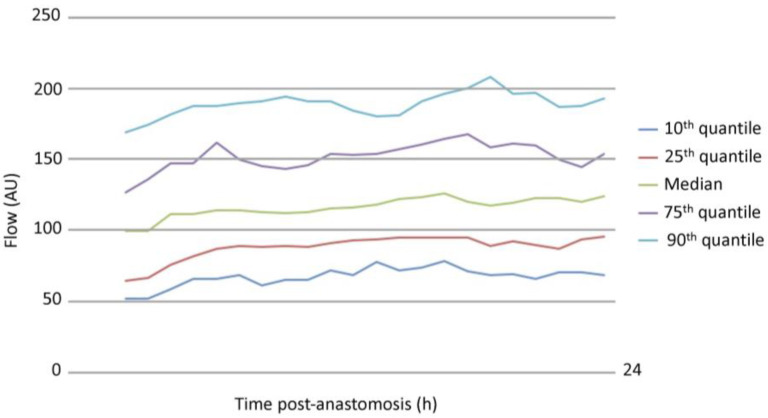
Microvascular flow quantiles over time.

Figure 1 displays a detailed view of the development of microvascular flow, sBP, and NIR over time. Inverse trends were observed for flow and NIR. While flow increased, NIR for cardiovascular support decreased, and sBP maintained stable values. Quantiles of flow over time are depicted in Figure 2.

### 3.2. Multivariate Analysis of Variables Potentially Affecting Microvascular Flow Post-Anastomosis

**Figure 3:**
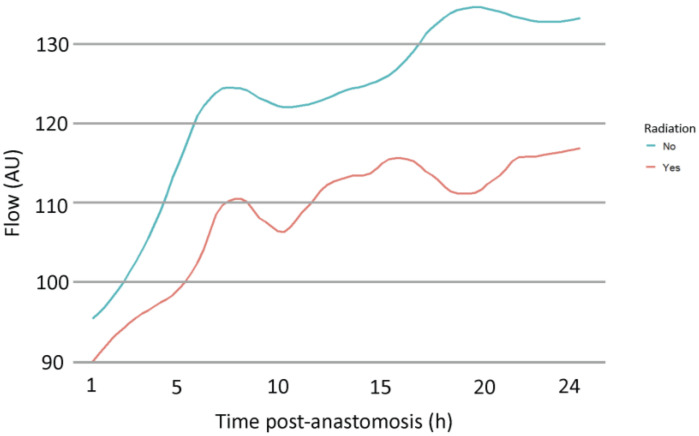
Development of microvascular flow in irradiated vs. non-irradiated patients over 24 h post-anastomosis.

Next, variables potentially affecting microvascular flow were investigated using multivariate analysis (Table 4). The coefficient of determination (R^2^) was 0.772, and the AIC was 3735. Therefore, 77.2% of changes in flow were predicted by the variables included in this model. Time, rHb, SO_2_, no prior radiation therapy, and female gender showed significant regression coefficients. Flow values increased with time (B = 3.76, *p* < 0.001), SO_2_ (B = 0.55, *p* < 0.001), female gender (B = 29.25, *p* = 0.02), and without previous radiation therapy (B = 41.21, *p* = 0.04), whereas they decreased with higher rHb values (B = −0.79, *p* < 0.001). Using the proposed regression model, the norepinephrine rates, sBP, smoking status, old age, and ASA score showed no significant impact on microvascular flow. No prior radiation therapy showed the highest regression coefficient (B = 41.21, *p* = 0.04) (Figure 3).

### 3.3. Subgroup Analysis According to sBP

**Figure 4:**
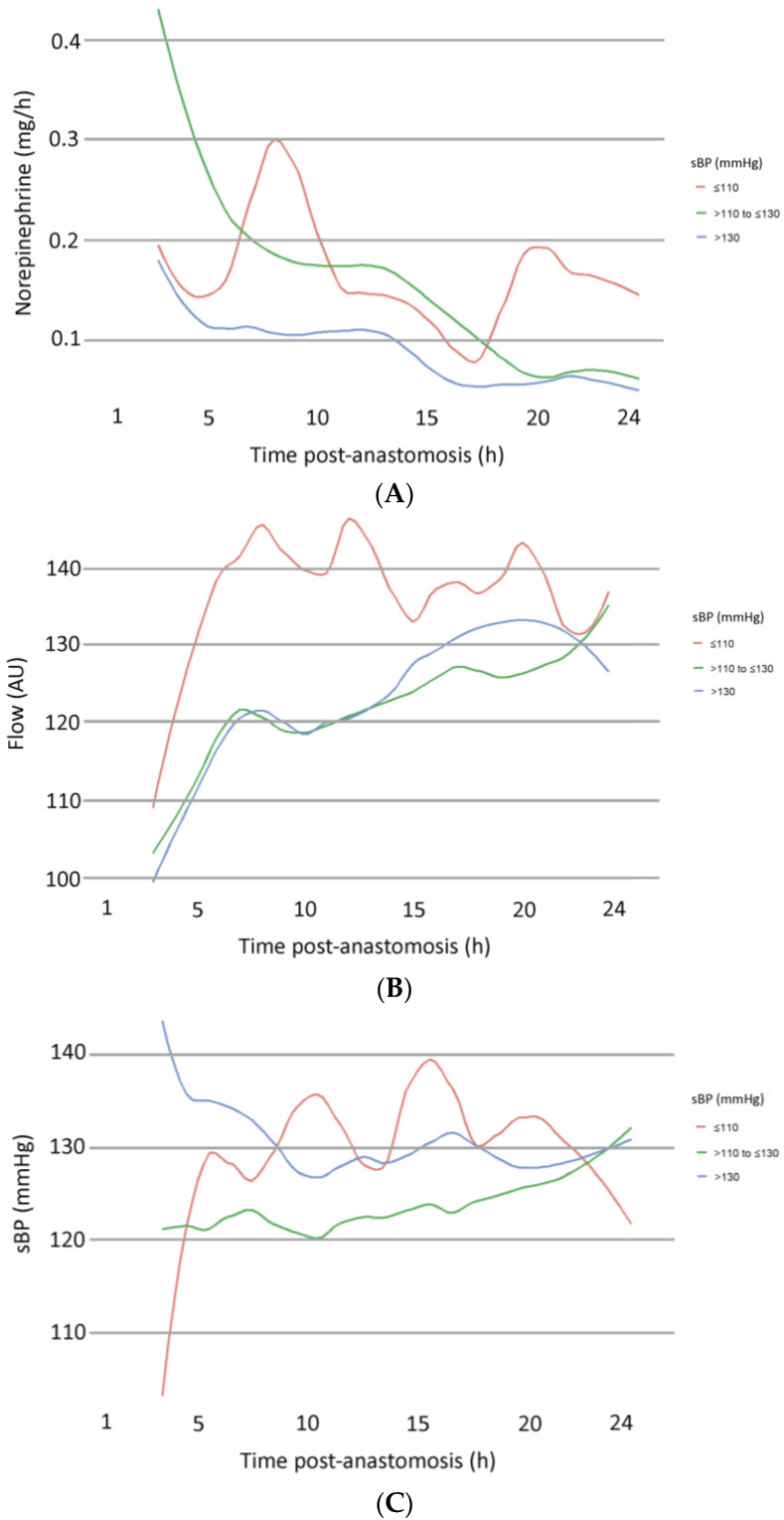
Subgroup analysis according to initial blood pressure values (low sBP, intermediate sBP, high sBP) of the development of norepinephrine infusion rates (**A**), microvascular flow (**B**), and systolic blood pressure (**C**).

The development of NIR, sBP, and microvascular flow was analyzed for the three subgroups (low-, intermediate-, and high-sBP), respectively (Figure 4). The following trends were observed: to compensate for low sBP, norepinephrine infusion rates showed an initial increasing trend in this subgroup, while a decrease was found in the intermediate and high sBP groups. This was reflected in a strong initial increase in sBP in the low sBP groups. Microvascular flow showed a steeper increase in patients with an initially low sBP compared to the intermediate and high sBP groups, matching the increase in norepinephrine and sBP in the early postoperative period. Multivariate analysis was performed to detect the significance of any of the graphically observed trends. However, no significant impact on microvascular flow was found for NIR or sBP in any subgroups (Table 5). Significance was once again shown for time, SO_2_, and rHb.

## 4. Discussion

The use of vasopressors in free-flap surgery has raised enduring concerns owing to the risk of inducing vasoconstriction and, subsequently, potentially jeopardizing flap viability. Over the past years, several systematic reviews have revoked this hypothesis by demonstrating that flap outcome was not inferior in patients receiving vasopressors while decreasing risks associated with fluid overload [7,12,13]. Henceforth, perioperative anesthesiologic management of patients requiring cardiovascular support during free flap surgery has shifted toward conservative fluid management and the utilization of vasopressors to manage hypotension [14]. The aim of this study was to provide further objective and quantitative data to support this clinical approach in a patient population requiring postoperative cardiovascular support after free flap surgery. Hence, this study investigated the impact of postoperative NIR and subsequent changes in patients’ sBP on the microvascular flow of free flaps. Importantly, the multivariate model explored in this study revealed no impact of NIR on free flap microcirculation, thus further underlining the safety of cardiovascular support via vasopressors in these patients. Further analysis was performed for subgroups with low-, intermediate-, and high-initial sBP values upon administration to the recovery room (IMC/ICU). Evidently, trend lines showed some differences. Exemplary, higher NIR were utilized for patients within the low sBP group in order to stabilize blood pressure. However, multivariate analysis revealed that these differences had no significant impact on the microvascular flow of free flaps, reinforcing the study’s key findings.

Most previous studies investigating the impact of blood pressure and vasopressors on free flaps focused on flap outcomes rather than truly measuring free flap microcirculation. A study performed by Eley et al. is an exception wherein the study group performed interrupted postoperative short-term measurements of blood flow in free flaps after administering different vasopressors using laser-doppler red blood cell velocimetry probes (Transonic Systems Inc., Ithaca, N.Y.) [15]. They concluded that norepinephrine elevated mean arterial pressure and caused an increase in blood flow of the flap, making it the ideal vasopressor for perioperative management of patients receiving free flaps [15]. Lee et al. provided data on the influence of norepinephrine and phenylephrine on blood flow in free transverse rectus abdominis myocutaneous flap breast reconstructions [16]. Utilizing transit time flowmetry, their data show a significant flow reduction in the phenylephrine group, while values changed non-significantly in the norepinephrine group. We hereby present a study that adds further objective and continuous, uninterrupted real-time data on microvascular flow, SO_2_, and rHb to assess the evolvement of microcirculation in free flaps, considering the changes in macrocirculation caused by norepinephrine and patients’ sBP. We regard this fact as a major strength of this study, adding further valuable literature to support the insignificant impact of common perioperative vasopressor use on microvascular flow.

Similar to previously published data, mean values of microvascular flow in free flaps showed a significant increase within the first 24 h after anastomosis [9,10,11], while norepinephrine rates decreased as intrinsic hemodynamic stability of the patient was re-established over time. The representation of flow quantiles in Figure 2 shows a large spread of values. These findings allow plastic surgeons to determine which values can be considered conspicuously low or high during postoperative flap monitoring, bearing in mind that all presented flaps included in this study showed no major complications, including no revision surgery and no vascular compromise. The large spread of flow values is likely related to the different types of free flaps included, as previous investigations determined different perfusion patterns in relation to specific flap types [9,17,18]. The increase in microvascular flow in the early postoperative period is most likely related to a decrease in vascular resistance caused by denervation of the tissue [19], in addition to reactive hyperemia following flap hypoxia [20].

Several potential confounding factors were included in the multivariate analysis of our study. Smoking has been thoroughly investigated as a risk factor for flap failure and complications, with most studies indicating safe flap surgery irrespective of smoking status [21,22,23]. In terms of changes in microcirculation, Booi et al. found lower blood-flow values in zone IV of free TRAM flaps in breast reconstructions [24], whereas Ooms et al. paradoxically found increased flow in heavy smokers receiving free flaps [25]. In our study population, multivariate analysis found no significant impact of smoking on microvascular flow.

The impact of preoperative radiation on free flap outcome is controversially debated [26]. Radiation injury alters the architecture of tissue and vessels [27], increasing the complexity of microsurgical reconstruction [28,29,30]. Foerster et al. investigated the impact of previous radiation therapy on blood flow in the recipient bed and transplanted free flaps in a population receiving head- and neck reconstruction. Their study group found reduced values for flow on the fifth and seventh postoperative day for the recipient bed, whereas no differences were found for the transplanted free flap [31]. In contrast to these findings, our study demonstrated lower flow values in flaps of irradiated patients. In addition, the multivariate model predicts significantly higher flow values for patients with no prior radiation therapy.

Numerous study groups have investigated the potential of the O2C device to detect vascular compromise. The data of this study showed an inverse relationship between rHb and microvascular flow, which has been previously related to venous congestion [32]. Furthermore, the analysis revealed a positive relationship between SO_2_ and flow. Consequently, the reduction in SO_2_ may predict a reduction in microvascular flow, which has previously been related to arterial occlusion [32]. Hence, objective and continuous real-time monitoring of free flap microcirculation may improve timely recognition of vascular compromise or hematoma causing pedicle compression and allow early emergent revision surgery to improve flap salvage rates. Future randomized controlled studies will need to evaluate the number of revision surgeries and free flap salvage rates in patients that were monitored with and without the use of the O2C device to further elucidate this hypothesis.

Most patients in the investigated study cohort had severe systemic disease, as reflected by the preoperative ASA classification. No significant impact of preoperative ASA score on microvascular flow was found. However, a closer evaluation of distinct comorbidities, especially vascular disease, might have elicited significant effects, as the recent literature points toward compromised flow measurements in patients with atherosclerotic vascular disease, although not persisting multivariable analysis [33]. This factor should be further investigated, especially in a cohort of extremity reconstructions.

### Limitations

Although many patients require cardiovascular support intraoperatively and during the early postoperative period in the recovery room, this study included only patients who were monitored at least until the first morning post-surgery to ensure data sufficiency for analysis. The morbidity of patients requiring postoperative administration to the IMC/ICU is reflected by the preoperative ASA classification (ASA 3: n = 70, 66.67%), most patients therefore displaying severe systemic disease. Hence, mean hospitalization was high with 17.19 ± 7.84 days after free flap transfer when compared with days to discharge of highly elective free flap transfers [34]. Due to the long length of stay, our study population could be considered a limitation. The study population was inhomogeneous regarding the free flap type utilized for defect reconstruction, defect location, and recipient vessel. The LFx37 probe (LEA Medizintechnik, Gießen, Germany) utilized is limited by measurements of microcirculation in approximately 7 mm depth measured from the free flap surface, irrespective of the type of free flap or flap tissue thickness, therefore not measuring specifically to a tissue microcirculatory network. In most patients, low-dose NIRs were utilized during postoperative care, and the range of sBP values evaluated was limited to three groups. Hence, the impact of peak values and spikes in patients’ sBP, as well as norepinephrine rates, should be further investigated in future studies.

While the multivariate analysis of our study included several confounding factors, other patient characteristics might also impact microvascular flow. Including additional parameters such as cardiac output, heart rate, blood gas analysis, and so on would further enhance the value of the presented analysis. Further subgroup analyses would also add value to the manuscript, and future research could focus on stratifying patients by other physiological parameters rather than sBP to offer a more comprehensive understanding of microcirculation outcomes. However, data extraction proved challenging as patient data are digitally archived in various data management systems. While the results of this study demonstrate that patient-related factors, such as previous radiation therapy, may impact the timely development as well as baseline values of microvascular flow, further research is warranted to evaluate the complex interplay between microcirculation, patient-related and perioperative factors after free tissue transfer, and to define threshold values for each parameter of microcirculation according to the individual patient profile.

## 5. Conclusions

Norepinephrine administration and subsequent changes in sBP in the early postoperative period after free flap reconstruction had no significant effect on free flap flow. These findings support the safe use of low-dose norepinephrine for maintaining stable systolic blood pressure without compromising microvascular flow, offering valuable guidance for plastic surgeons and anesthesiologists in postoperative management.

## Figures and Tables

**Table 1:** Demographic variables and patient characteristics.

Variable	
Age (y), mean ± SD	61.46 ± 16.29
Gender, n (%)	
Male	50 (47.62%)
Female	55 (52.38%)
BMI (kg/m^2^), mean ± SD	25.11 ± 5.76
ASA classification, n (%)	
1	3 (2.86%)
2	23 (21.90%)
3	70 (66.67%)
4	9 (8.57%)
Co-morbidities, n (%)	
Hypertension	71 (67.62%)
PAD	9 (8.57%)
Diabetes	21 (20%)
History of thrombosis	23 (21.90%)
History of myocardial infarction	20 (19.05%)
Hospitalization (d), mean ± SD	17.19 ± 7.84
Days on IMC/ICU	3.97 ± 12.97
Defect etiology, n (%)	
Tumor	57 (54.29%)
Trauma	14 (13.33%)
Infection	12 (11.43%)
Chronic wound	16 (15.24%)
Other	6 (5.71%)
Defect location, n (%)	
Head and neck	36 (34.29%)
Trunk	32 (30.48%)
Upper extremity	14 (13.33%)
Lower extremity	23 (21.90%)
Previous radiation therapy, n (%)	
Yes	29 (27.62%)
No	76 (72.38%)
Smoker	
Yes	18 (17.14%)
No	10 (9.52%)
Ex	11 (10.48%)
NR	66 (62.86%)

D = days; n = number; NR = not reported; PAD = peripheral arterial disease; SD = standard deviation; y = years.

**Table 2:** Operative details of study population.

Variable	
Type of free flap, n (%)	
GM	15 (14.29%)
LDM	37 (35.24%)
ALT/MVL	34 (32.38%)
DIEP	10 (9.52%)
Others	9 (8.57%)
Time of surgery (min), mean ± SD	354.42 ± 110.58
Flap ischemia (min), mean ± SD	48.66 ± 20.39
Recipient vessel, n (%)	
Internal mammary	22 (20.95%)
Superior thyroid	10 (9.52%)
Temporal	19 (18.10%)
Facial	10 (9.52%)
Anterior tibial	8 (7.62%)
Posterior tibial	8 (7.62%)
Fibular	2 (1.90%)
Radial	5 (4.76%)
Ulnar	4 (3.81%)
Others	17 (16.19%)
Number of venous anastomoses, n (%)	
1	69 (65.71%)
2	36 (34.29%)

ALT = anterolateral thigh; DIEP = deep inferior epigastric artery perforator; GM = gracilis muscle; LDM = latissimus dorsi muscle; min = minutes; MVL = myocutaneous vastus lateralis; n= number; SD = standard deviation.

**Table 3:** Descriptive analysis of microcirculation in free flaps up to 24 h post-anastomosis. Pairwise comparisons across time points were performed for each variable using the Wilcoxon test. *p*-values refer to comparisons with the respective value at 3 h post-anastomosis. Values are presented as means (standard deviation).

Hour	Flow (AU)	*p*-Value	SO_2_ (%)	*p*-Value	rHb (AU)	*p*-Value	MAD (mmHg)	*p*-Value	sBP (mmHg)	*p*-Value	dBP (mmHg)	*p*-Value	Norepinephrine (mg/h)	*p*-Value
3	104.27 (44.85)		43.62 (22.51)		37.30 (12.90)		85.14 (9.64)		128.36 (15.26)		61.67 (8.44)		0.28 (0.37)	
4	106.63 (46.39)	1	42.65 (21.95)	1	37.91 (13.67)	1	83.66 (10.77)	1	127.20 (16.92)	1	60.63 (9.28)	1	0.24 (0.37)	0.34
5	113.96 (47.72)	0.37	41.51 (22.02)	1	36.16 (12.01)	1	85.02 (10.79)	1	129.07 (17.13)	1	61.09 (9.15)	1	0.20 (0.37)	0.02
6	118.46 (48.47)	0.48	42.80 (21.10)	1	37.28 (12.53)	1	84.21 (11.01)	1	129.24 (16.25)	1	60.39 (10.08)	1	0.16 (0.29)	0.02
7	122.58 (49.32)	0.12	43.34 (20.57)	1	36.56 (12.43)	1	82.75 (11.71)	1	127.60 (16.59)	1	59.33 (10.34)	1	0.16 (0.25)	0.04
8	121.96 (48.38)	0.02	43.44 (20.98)	1	36.16 (11.95)	1	82.93 (10.19)	1	128.30 (16.72)	1	59.43 (8.66)	1	0.14 (0.20)	0.01
9	120.98 (49.46)	0.12	42.45 (20.38)	1	35.811 (12.39)	1	79.06 (10.20)	0.05	124.08 (15.17)	1	56.65 (9.42)	<0.001	0.14 (0.21)	0.02
10	120.28 (49.28)	0.04	41.67 (20.43)	1	35.88 (12.52)	1	81.06 (10.65)	0.96	125.76 (15.86)	1	57.87 (9.48)	0.07	0.14 (0.20)	0.05
11	120.46 (48.51)	0.01	41.55 (20.27)	1	35.96 (12.10)	1	80.39 (10.13)	0.25	125.92 (16.37)	1	56.44 (8.25)	<0.001	0.14 (0.19)	0.03
12	123.49 (47.74)	<0.001	41.51 (20.56)	1	35.43 (12.19)	1	80.68 (9.87)	1	126.88 (17.93)	1	56.81 (8.41)	0.01	0.14 (0.12)	0.05
13	123.04 (45.79)	<0.001	41.60 (20.57)	1	35.54 (12.41)	1	80.04 (8.77)	0.15	126.23 (15.10)	1	56.11 (7.60)	<0.001	0.13 (0.19)	0.03
**Hour**	**Flow (AU)**	***p*** **-value**	**SO_2_ (%)**	***p*** **-value**	**rHb (AU)**	***p*** **-value**	**MBP (mmHg)**	***p*** **-value**	**sBP (mmHg)**	***p*** **-value**	**dBP (mmHg)**	***p*** **-value**	**Norepinephrine (mg/h)**	***p*** **-value**
14	124.20 (44.71)	<0.001	40.98 (21.14)	1	35.84 (12.77)	1	79.48 (9.26)	0.11	125.56 (14.95)	1	55.58 (8.66)	<0.001	0.12 (0.17)	0.01
15	125.28 (45.12)	<0.001	41.48 (20.87)	1	36.08 (12.99)	1	84.16 (21.20)	1	130.52 (22.42)	1	59.91 (23.19)	0.37	0.11 (0.16)	<0.001
16	127.44 (47.34)	<0.001	41.94 (20.68)	1	36.49 (12.80)	1	80.33 (10.16)	0.25	127.00 (18.51)	1	56.68 (8.27)	<0.001	0.09 (0.15)	<0.001
17	130.09 (48.03)	<0.001	42.40 (21.47)	1	35.82 (12.67)	1	82.85 (11.89)	1	129.16 (19.36)	1	58.22 (10.63)	0.69	0.08 (0.16)	<0.001
18	130.45 (51.92)	<0.001	42.49 (22.25)	1	35.60 (12.45)	1	84.95 (10.86)	1	129.29 (18.15)	1	59.39 (8.88)	1	0.08 (0.14)	<0.001
19	128.83 (54.34)	<0.001	40.53 (21.63)	1	36.23 (12.63)	1	82.06 (12.55)	1	127.22 (17.08)	1	58.26 (11.43)	1	0.06 (0.12)	<0.001
20	129.84 (54.59)	<0.001	37.87 (20.54)	1	36.26 (12.18)	1	83.19 (15.12)	1	129.27 (20.51)	1	57.70 (12.35)	1	0.06 (0.13)	<0.001
21	129.83 (54.00)	<0.001	38.86 (20.71)	1	37.19 (14.15)	1	82.03 (11.14)	1	130.55 (17.22)	1	57.61 (10.30)	1	0.07 (0.16)	<0.001
22	128.31 (60.08)	<0.001	40.01 (22.46)	1	39.18 (19.42)	1	81.37 (11.37)	1	126.15 (16.60)	1	58.93 (12.10)	1	0.07 (0.17)	<0.001
23	129.15 (56.76)	<0.001	40.01 (21.45)	1	39.59 (19.04)	1	83.40 (16.10)	1	131.75 (21.36)	1	60.65 (15.86)	1	0.06 (0.17)	<0.001
24	131.63 (59.06)	<0.001	39.36 (21.43)	1	37.59 (12.26)	1	81.70 (11.80)	1	128.87 (15.48)	1	58.55 (11.92)	1	0.06 (0.18)	<0.001

AU = arbitrary units; dBP = diastolic blood pressure; MBP = mean blood pressure; sBP = systolic blood pressure.

**Table 4:** Multivariate analysis of variables predicting microvascular flow after free flap transfer.

	Value	Std. Error	DF	t-Value	*p*-Value
(Intercept)	43.27	23.78	413	1.82	0.07
rHb	−0.79	0.16	413	−5.04	<0.001
SO_2_	0.55	0.13	413	4.27	<0.001
Smoker no	21.39	15.69	26	1.36	0.18
Smoker ex	2.5	14.76	26	0.17	0.87
ASA L	−28.56	22.78	26	−1.25	0.22
ASA Q	12.34	17.11	26	0.72	0.48
ASA C	−1.1	13.27	26	−0.08	0.93
Norepinephrine	6.54	9.91	413	0.66	0.51
sBP	0.06	0.05	413	1.11	0.27
Time (h)	3.76	1.24	413	3.03	<0.001
Time_quad	−0.11	0.05	413	−2.16	0.03
Age > 65	−9.19	12.88	26	−0.71	0.48
Gender female	29.25	11.78	26	2.48	0.02
Radiation no	41.21	18.53	26	2.22	0.04

h = hour, sBP = systolic blood pressure.

**Table 5:** Multivariate analysis predicting the effects of norepinephrine infusion rates and subgroups of systolic blood pressure on microvascular flow.

	Value	Std. Error	DF	t-Value	*p*-Value
(Intercept)	99.156	15.823	432.000	6.267	<0.001
rHb	−0.396	0.162	432.000	−2.440	0.015
SO_2_	0.307	0.105	432.000	2.919	0.004
Smoker	1.900	4.358	87.000	0.436	0.664
ASA 3–4	−19.859	10.439	87.000	−1.902	0.060
Norepinephrine	3.998	5.992	432.000	0.667	0.505
sBP	0.013	0.058	432.000	0.219	0.827
Time (h)	9.333	3.468	432.000	2.691	0.007
Time_quad	−0.368	0.241	432.000	−1.525	0.128
sBP >110 to ≤130	−2.251	1.946	432.000	−1.157	0.248
sBP > 130	−2.754	1.959	432.000	−1.406	0.160

h = hour, sBP = systolic blood pressure.

## Data Availability

The original contributions presented in this study are included in the article. Further inquiries can be directed to the corresponding author(s).
